# Human metapneumovirus as cause of severe community-acquired pneumonia in adults: insights from a ten-year molecular and epidemiological analysis

**DOI:** 10.1186/s13613-019-0559-y

**Published:** 2019-07-24

**Authors:** Loreto Vidaur, Izarne Totorika, Milagrosa Montes, Diego Vicente, Jordi Rello, Gustavo Cilla

**Affiliations:** 1 Critical Care Department, Donostia University Hospital-Biodonostia Health Research Institute, San Sebastian, Guipuzcoa Spain; 2 Microbiology Department, Donostia University Hospital-Biodonostia Health Research Institute, San Sebastian, Guipuzcoa, Spain; 3 CIBERES, Institute of Health Carlos III, Madrid, Spain; 4 Faculty of Medicine, University of Basque Country (UPV/EHU), San Sebastian, Guipuzcoa Spain; 5 Research Institute Vall d`Hebron University Hospital (VHIR), Barcelona, Spain

**Keywords:** Severe community-acquired pneumonia, Human metapneumovirus, Acute respiratory distress syndrome, Biomarkers

## Abstract

**Background:**

Information on the clinical, epidemiological and molecular characterization of human metapneumovirus in critically ill adult patients with severe community-acquired pneumonia (CAP) and the role of biomarkers identifying bacterial coinfection is scarce.

**Methods:**

This is a retrospective epidemiological study of adult patients with hMPV severe CAP admitted to ICU during a ten-year period with admission PSI score ≥ 3.

**Results:**

The 92.8% of the 28 patients with severe CAP due to human metapneumovirus were detected during the first half of the year. Median age was 62 years and 60.7% were male. The genotyping of isolated human metapneumovirus showed group B predominance (60.7%). All patients had acute respiratory failure. Median APACHE II and SOFA score were 13 and 6.55, respectively. The 25% were coinfected with *Streptococcus pneumoniae.* 60.7% of the patients had shock at admission and 50% underwent mechanical ventilation. Seven patients developed ARDS, three of them younger than 60 years and without comorbidities. Mortality in ICU was 14.3%. Among survivors, ICU and hospital stay were 6.5 and 14 days, respectively. Plasma levels of procalcitonin were higher in patients with bacterial coinfection (18.2 vs 0.54; *p* < 0.05). The levels of C-reactive protein, however, were similar.

**Conclusion:**

Human metapneumovirus was associated with severe CAP requiring ICU admission among elderly patients or patients with comorbidities, but also in healthy young subjects. These patients often underwent mechanical ventilation with elevated health resource consumption. While one out of four patients showed pneumococcal coinfection, plasma procalcitonin helped to implement antimicrobial stewardship.

**Electronic supplementary material:**

The online version of this article (10.1186/s13613-019-0559-y) contains supplementary material, which is available to authorized users.

## Background

Human metapneumovirus (hMPV) is a worldwide distributed enveloped virus with a RNA genome closely related to respiratory syncytial virus. hMPV belongs to the *Paramyxoviridae* family, in the genus *Metapneumovirus*, first identified in the Netherlands in 2001 [1]. Based on genetic and antigenic variability, hMPV strains have been classified in two groups or lineages (A and B) and four sublineages (A1, A2, B1 and B2) [1–3]. The virus has been reported as a common respiratory pathogen in childhood, associated mainly with upper but also with lower respiratory tract infections [2, 4]. During the annual epidemics, hMPV has been associated with a significant number of hospital admissions in young children [4–7].

Respiratory tract infections caused by hMPV during adulthood are less prevalent and less serious than those in childhood. However, the presence of hMPV has been detected in 2–4% of adult patients admitted due to a community-acquired pneumonia (CAP) [8, 9] and has been associated with asthma and chronic obstructive pulmonary disease exacerbation [10–12]. The same as with other common respiratory viruses, hMPV is usually associated with non-severe pneumonia, whereas risk factors like immunosuppression, specific comorbidities—chronic lung disease, heart disease, blood disorders— elderly and living in long-term care facilities are associated with a higher risk of severe viral pneumonia [13, 14]. Nevertheless, recent studies suggest that hMPV infection is an underappreciated cause of critical illness, also in previously healthy patients [15–18].

Severe community-acquired pneumonia (SCAP) is a known infectious complication of respiratory viruses including hMPV. In these cases, clinical presentation, evolution and treatment differ depending on the pathogens involved, hMPV alone or hMPV coinfected with a bacteria. Some biomarkers have been studied as diagnostic markers to discriminate between viral or bacterial pneumonias and help physicians to decide not to start or when to withdraw the antibiotic therapy [19, 20].

The main objective of this study was to describe the clinical and epidemiological characteristics of adults with severe pneumonia caused by hMPV who required intensive care unit (ICU) admission, over a long period of time. Secondary objectives were to characterize the epidemiological and molecular viral diversity and to compare the value of C-reactive protein (CRP) and procalcitonin in identifying bacterial coinfections.

## Methods

This is a ten-year, retrospective epidemiological study with inclusion of patients with CAP due to hMPV admitted in a 48-bed ICU in the North of Spain. In 2017, this ICU assisted a referral population of 545,227 inhabitants older than 14 years.

All patients older than 14 years from July 2007 to June 2017 admitted in the ICU by CAP with admission PSI score ≥ 3 were considered eligible. During the first 2 years of the study, samples to detect respiratory viruses were obtained occasionally in patients with CAP. However, it turned the standard of care in the ICU after 2009 influenza pandemic. To be included, cases meet two of the following three criteria upon admission: (a) severe acute respiratory failure (paO2/FiO2 < 250), (b) multilobar radiological involvement or (c) systolic arterial pressure < 90 mmHg. Acute respiratory distress syndrome (ARDS) was diagnosed as an acute diffuse lung injury with increased vascular permeability, bilateral radiographic opacities and hypoxemia not fully explained by cardiac failure or fluid overload following the Berlin criteria [21]. Exclusion criteria: subjects with nosocomial pneumonia or admitted due to non-respiratory infection (non-severe coincidental infection) and patients with pneumonia during the preceding 2 months (persistence of viral RNA in respiratory samples). Patients were recruited from the computerized records of the Microbiology department, and the medical records were revised by two clinical investigators (IT, LV). The recorded clinical variables were socio-demographic (age and sex), comorbidities, the Charlson comorbidity score and clinical symptoms at admission [22]. Radiological and analytic findings at admission and during the evolution, coinfections, antibiotic therapy, the presence of shock or need of mechanical ventilation, ICU and hospital stay were also recorded.

The detection of hMPV in respiratory samples was made by reverse transcription polymerase chain reaction (RT-PCR), in house monoplex until July 2010 [5], real-time commercial multiplex (Luminex xTAG Respiratory viral panel [USA]) until July 2013 and Seegene Anyplex™ II RV16/Allplex™ Respiratory Panel [Republic of Korea] since then. The extraction of nucleic acids was made using an automatic BioRobot1 M48 extractor (Qiagen GmbH, Hilden, Germany) until July 2009 and the NucliSENS^®^ Easy-Mag platform (bio-Mèrieux SA, Marcy l’Etoile, France) from that date. The genotyping of hMPV was performed with a RT-PCR followed by sequencing [23]. Blood cultures, *Streptococcus pneumoniae* and *Legionella pneumophila* antigenuria (Alere BinaxNOW, Scarborough, ME, USA), and pharyngeal exudates with viral transport media to evaluate respiratory viruses were assessed in all the patients included in the study. Coinfection was considered when hMPV was isolated with other viral or bacterial pathogens at the same time.

Discrete variables were expressed as counts (percentage) and continuous variables as medians and 25–75% interquartile ranges (IQRs). Differences in continuous variables were analyzed by the Mann–Whitney *U* test or the Kruskall–Wallis test when appropriate. Qualitative variables were analyzed by the Chi-square test with Yate’s correction when necessary. The threshold for clinical significance was *p* < 0.05. Data analysis was performed using SPSS for Windows 21.0.0 (SPSS, Chicago, IL, USA). The obtained clinical samples and the medical intervention of the patients were ordered by the clinician attending each patient. The study was approved by the ethics committee for clinical research of the health area of Gipuzkoa (Spain). Informed consent was waived due to the retrospective nature of the study.

## Results

**Fig. 1:**
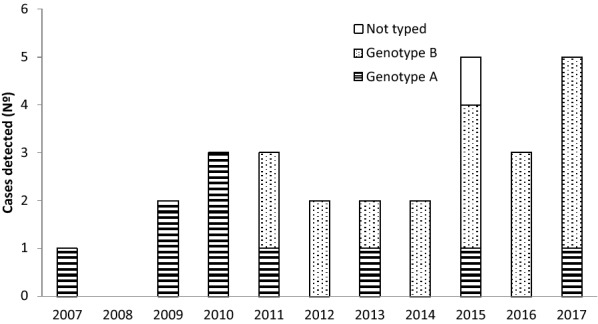
Cases of severe community-acquired pneumonia associated with human metapneumovirus infection, hospitalized in the Intensive Care Unit of a large regional hospital (San Sebastián, North of Spain)

During the study period, 1942 respiratory samples were sent from the ICU to the Microbiology Service to study viral etiology, hMPV being identified in 33 patients (1.7%). Studied samples were mainly pharyngeal exudates (77.3%), but also tracheal aspirates (7.5%), bronchoaspirates (5.8%), bronchoalveolar lavages (5.6%) and sputum (4.2%), where bacterial culture was also performed. Five patients with hMPV were excluded because admission causes were other than respiratory infection. Cases were detected every year except in 2008 (*n* = 0–5). The highest prevalence was in 2015 and 2017 (Fig. 1). Twenty-six of the 28 patients with respiratory infection due to hMPV (92.9%) were detected during the first half of the year and 16 (57.1%) in March–April. HMPV circulated every year later than influenza virus, being the epidemic peak of both infections separated by a period of 1–2 months. In fact, the 75% of cases (21/28) of hMPV infections in patients admitted to ICU occurred out of the influenza epidemic period (Table 1).

**Table 1:** Annual epidemic peaks of influenza virus and human metapneumovirus infections, and temporal distribution of isolates in patients with severe community-acquired pneumonia due to human metapneumovirus infection (Gipuzkoa, Spain, 2008–2017)

Year	Flu epidemic peak	hMPV epidemic peak	SCAP hMPV
2008	January (2nd week)	March	None
2009	December (2008) (50th week)	January	April (*n* = 1) July (*n* = 1)
2010	October (2009) Pandemia (42th week)	March	March (*n* = 3)
2011	January (1st week)	March	March (*n* = 2) April (*n* = 1)
2012	February (7th week)	March	June (*n* = 2)
2013	February (7th week)	March	May (*n* = 1) July (*n* = 1)
2014	January (3rd week)	March	January 2014 (*n* = 1) April 2014 (*n* = 1)
2015	February (5th week)	March	January (*n* = 1) March (*n* = 1) April (*n* = 2) May (*n* = 1)
2016	January (4th week)	March	March (*n* = 3)
2017	January (3rd week)	March	January (*n* = 2) February (*n* = 1) March (*n* = 2)

*hMPV* human metapneumovirus, *SCAP* severe community-acquired pneumonia

Genotyping of hMPV was performed in 27 cases, being ten cases of hMPV group A (39.3%) and 17 of hMPV group B (60.7%). The viral strains belonged to sublineages A2 (*n* = 10; 39.3%), B1 (*n* = 11; 39.3%) and B2 (*n* = 6; 21.4%). Group A strains predominated until 2011 (72.7%), while later, the most frequent was genotype B (79%). After excluding seven patients with bacterial coinfection, there were not significant differences in the genotype of hMPV between six patients who developed ARDS (60% genotype B) and 15 who did not (67% genotype B).

At ICU admission, all patients had acute respiratory failure and received empiric antibiotic therapy. Median APACHE II score was 13 [IQR 11.2–20], and median SAPS III and SOFA scores were 58.5 [IQR 47.2–70.7] and 6.5 [IQR 3.5–9.5], respectively. Median age of the included patients was 62 years [25–75% IQR 49.7–75.7], and 60.7% of them were under 65 years old (9 with less than two comorbidities). The 60.7% (*n* = 17) of the patients were male. Main symptoms at admission were cough (89.3%), dyspnea (71.4%), fever (67.9%) and purulent respiratory secretions (67.8%). Nineteen patients (67.9%) had major comorbidities such as immune compromise (*n* = 7), asthma (*n* = 3) or chronic respiratory disease (*n* = 3) (Table 2). Seven patients (none died) had coinfection with *Streptococcus pneumoniae*. Three episodes were coinfected with viral pathogens: human parainfluenza virus type 3 (hPIV3), human rhinovirus and cytomegalovirus (last one in an immunosuppressed patient).

**Table 2:** Demographic characteristics of the study population

	Total *n* (%)	Bacterial coinfection (*n*)	Without bacterial coinfection (*n*)
Study patients (*n*)	28 (100)	7	21
Male	17 (60.7)	5	12
Age (years) median [ICR]	62 [50 to 76]	62 [61 to 83]	62 [48 to 75]
Comorbidities			
COPD	3 (10.7)	1	2
Asthma	3 (10.7)	0	3
Heart failure	2 (7.1)	1	1
Alcoholism	3 (10.7)	0	3
Immunosuppression	7 (25.0)	2	5
No comorbidities	11 (39.3)	3	8
Clinical manifestations			
Fever	19 (67.9)	6	13
Cough	25 (89.3)	6	19
Purulent secretions	19 (67.9)	5	14
Dyspnea	20 (71.4)	5	15
Radiologic pattern			
Alveolar	15 (53.6)	6	9
Interstitial	3 (10.7)	1	2
Interstitial alveolar	10 (35.7)	0	10
Bilateral	17 (60.7)	2	15
Pleural effusion	8 (28.6)	2	6
Complete blood count			
Leukopenia/leukocytosis/mm^3^ (< 4000 year > 11,000)	16 (57.1)	4	12
Thrombocytopenia/mm^3^ (< 150,000)	9 (32.1)	4	5
Lymphopenia/mm^3^ (< 1000)	23 (82.1)	6	17

*COPD* chronic obstructive pulmonary disease

Predominant radiologic pattern in patients with hMPV infection and without coinfection was the interstitial alveolar pattern (47.6%), while in the patients with *Streptococcus pneumoniae* coinfection, the alveolar pattern was predominant (85.7%). Eight patients had pleural effusion at admission, and two more developed it during the ICU stay. Pleural effusion was bilateral in four patients and massive (> 2 L) in three cases.

Seventeen (60.7%) patients had shock at admission, fourteen (50%) underwent invasive mechanical ventilation (median 5.5 days [IQR 5–14.2]) due to acute respiratory failure and four were tracheostomized due to prolonged mechanical ventilation. Severe complications were frequent, highlighting acute renal failure in 12 patients (42.8%), of which two required renal replacement therapy; cardiac failure or cardiogenic shock in eight patients (28.5%); and ARDS in seven cases (25%) (two of them in patients with bacterial coinfection) (Table 2). Three patients who developed ARDS were younger than 60 years (38, 47 and 54 years, respectively) without major comorbidities or bacterial coinfection. All of them underwent invasive mechanical ventilation due to acute respiratory failure (one had coinfection with hPIV3). The main clinical and epidemiological characteristics of the patients are summarized in the Supplementary material (Additional file 1: Table S1, Additional file 2: Table S2). The majority of the patients (83.3%) had lymphocytopenia (< 1000/mL) at admission (Table 2). Four patients (average age 68.5 years), three of them immunocompromised, died during the ICU stay due to complications of respiratory failure. Among survivors, ICU and hospital median stay were 6.5 [IQR 5–11.7] and 14 [IQR 10–23.3] days, respectively. Survival rate after hospital discharge at 3 and 6 months was 97.1% (*n* = 23) and 87.5% (*n* = 21), respectively.

The median CRP and procalcitonin plasma levels at ICU admission in the 28 hMPV-infected patients with CAP were 125 [44.6–305.9] mg/L and 1.3 [0.2–9.3] ng/mL, respectively. The values of CRP were similar between patients with bacterial coinfection and those without (median 231.4 [IQR 84.0–407.5] vs 110.2 [38–281.7] mg/L; *p* = 0.29). In contrast, the plasma levels of procalcitonin were higher in the patients with bacterial coinfection than in those without (median, 18.2 [IQR 8.4–57.6] vs 0.54 [0.1–1.9] ng/mL, *p* < 0.005). All patients with bacterial coinfection had procalcitonin > 1 ng/mL at ICU admission.

## Discussion

Our study gives new insights on the molecular epidemiology of hMPV pneumonia admitted to the ICU over 10 years. HMPV was consistently detected in CAP admitted to the ICU, with an annual incidence ranging 0.5–1 case/100,000 inhabitants older than 14 years per year. Molecular characterization of hMPV revealed group dominance of subgroup B. HMPV infection presented seasonal distribution, with 2/3 of cases detected in late winter-early spring each year. The 32% of the studied patients were younger than 65 years without comorbidities. HMPV CAP often presented as acute respiratory failure with bilateral opacities and half of ICU subjects underwent mechanical ventilation. Lymphocytopenia and pleural effusion were common at admission. Plasma procalcitonin was a sensitive tool to identify coinfection with bacteria (25%), which contributes to antimicrobial stewardship. These findings suggest the need to implement hMPV diagnosis tests in subjects with CAP developing acute respiratory failure.

Two out of three patients of this study had shock at admission, half of them underwent mechanical ventilation, one out of four developed ARDS and one out of seven died during the clinical course, suggesting that hMPV is responsible for SCAP in adults. These data are concordant to that observed in the only study with a wide range of patients with hMPV infection in critically ill patients, in which 55% of the patients required mechanical ventilation, 48% developed ARDS and the mortality was 18% [18]. Moreover, there are sporadic reports of 3–6 patients with hMPV infection acquired in the community and acute respiratory failure who required ICU admission [15–17]. In a large prospective study of ICU patients requiring invasive mechanical ventilation, hMPV was more frequently detected in patients admitted by severe respiratory infection than in patients with other causes, suggesting a causal role of HMPV in the development of severe respiratory infection [24].

Most of the patients of this study had major comorbidities at admission, mainly chronic respiratory failure and immunosuppression, being those patients and the elderly the most susceptible to develop severe hMPV infections [2, 10, 14]. However, 60% of the patients were younger than 65 years old and one out of three did not have major comorbidities, being similar to CAP related to other etiologies. Interestingly, three patients (10.7%) were young adult patients without comorbidities and without bacterial coinfection that developed ARDS pointing out a main role of hMPV in the etiology of severe respiratory infections requiring mechanical ventilation. In the cohort of patients of Hasvold et al. [18], 15% of the patients had only minor comorbidities and were not immunosuppressed.

One out of four episodes of severe acute respiratory infection was coinfected with bacteria, similar to that observed in other series [17, 18]. *Streptococcus pneumoniae*, one of the bacterial species most frequently involved in post-viral super-infections [25], was the main isolated bacterial pathogen. In these episodes, procalcitonin has been reported to discriminate between viral episodes and those with bacterial coinfection [26], in contrast with CRP. Some studies have recommended different cutoff points of procalcitonin to discontinue early antibiotic therapy in patients with community-acquired therapy, being 0.25 ng/mL and, mainly 0.1 ng/mL the most recommended [27, 28]. None of the patients with documented bacterial coinfection in this study had a procalcitonin level lower than 1 ng/mL which supports the early discontinuation of antibiotic therapy in this group of patients with low plasma levels of procalcitonin. The results of this study, about procalcitonin plasma determinations, could help to develop personalized medicine in patients with CAP, helping physicians to early discrimination between viral or bacterial pneumonia and antimicrobial stewardship [29].

Three different genotypes of hMPV were associated with severe CAP requiring ICU admission, which supports that all of them are able to cause severe infections in adult patients. The low number of cases of the three different hMPV lineages, the presence of coinfections and the retrospective nature of the study made impossible to analyze the clinical pattern and the evolution of the patients based on the genotype of the infecting hMPV. However, to date, there are no significant differences in the evolution or clinical manifestation between different genotypes of HMPV in adults in the outpatient setting [30].

This study has some limitations and therefore, the results should be evaluated cautiously. The hMPV infection was diagnosed by oropharyngeal swab samples more than in low respiratory tract samples, mainly in non-intubated patients. The detection of a viral pathogen in respiratory samples of a patient with acute respiratory infection can be coincident and not related to ICU admission. The retrospective design of the study can underestimate the actual incidence of hMPV infection because some patients admitted because of acute respiratory infection could not be investigated for viral etiology. However, from the 2009 influenza pandemics, nasopharyngeal swab samples with respiratory viral detection are routine of care being collected in the 90% of patients with SCAP admitted to ICU. Finally, three different molecular techniques were used, with potential selection bias due to the differences in sensitivity of these techniques.

## Conclusion

In conclusion, our study confirms that hMPV, a respiratory virus causing bronchiolitis and pneumonia in children, was associated with severe CAP requiring ICU admission among elderly patients or patients with comorbidities, but also in healthy young subjects. These patients often underwent mechanical ventilation with long ICU and hospital stays, associated with elevated health resource consumption. The results of this study agree with recent observations [13] suggesting a shift in the paradigm of severe pneumonia, recommending that viral infection (and specifically hMPV) should be ruled out when complicated with acute respiratory failure. While one out of four patients showed pneumococcal coinfection, plasma procalcitonin levels helped to implement antimicrobial stewardship.

## Additional files

**Additional file 1: Table S1.** Main characteristics of immunocompetent adult patients admitted to the Intensive Care Unit due to a severe community-acquired pneumonia associated with human metapneumovirus infection (Guipuzcoa, Basque Country, Spain, 2007–2017).

**Additional file 2: Table S2.** Main characteristics of immunosuppressed adult patients admitted to the Intensive Care Unit due to a severe community-acquired pneumonia associated with human metapneumovirus infection (Guipuzcoa, Basque Country, Spain, 2007–2017).

## Data Availability

The datasets supporting the conclusions of this article are included within the article (and its additional file).

## Abbreviations

ARDS: acute respiratory distress syndrome
APACHE II: acute physiology and chronic health evaluation II
CAP: community-acquired pneumonia
COPD: chronic obstructive pulmonary disease
CRP: C-reactive protein
hMPV: human metapneumovirus
ICU: intensive care unit
PSI Score: Pneumonia Severity Index
SAPS III: simplified acute physiology score III
SCAP: severe community-acquired pneumonia
SOFA: sequential organ failure assessment
