# Supplementary material for: Human metapneumovirus as cause of severe community-acquired pneumonia in adults: insights from a ten-year molecular and epidemiological analysis
Source: Ann Intensive Care. 2019 Jul 24;9:86. doi: 10.1186/s13613-019-0559-y (PMC6656825; doi:10.1186/s13613-019-0559-y)
Supplement: Supplementary file 1 — Additional file 1: Table S1. Main characteristics of immunocompetent adult patients admitted to the Intensive Care Unit due to a severe community-acquired pneumonia associated with human metapneumovirus infection (Guipuzcoa, Basque Country, Spain, 2007–2017). [file 13613_2019_559_MOESM1_ESM.docx]

Additional file 1: Table S1: Main characteristics of immunocompetent adult patients admitted to the Intensive Care Unit due to a Severe community-acquired pneumonia associated to human metapneumovirus infection (Guipuzcoa, Basque Country, Spain, 2007-2017)

| Age/Sex | Genotype | Comorbidities | APACHE II/SOFA | PCT(ng/ml)  CRP(mg/dl) | Coinfection | ARDS | Shock | Hospital Stay (ICU) | Survival at discharge |
| --- | --- | --- | --- | --- | --- | --- | --- | --- | --- |
| 62/M^1^ | A2B | COPD^3^ | 12/6 | <0,5/12,28 | No | No | Yes | 13(6) | Yes |
| 70/F^2^ | A2A | Asthma | 11/5 | 0,05/2,7 | No | No | Yes | 14(9) | Yes |
| 64/M^1^ | A2 |  | 8/5 | 1,8/452 | Rhinovirus | No | Yes | 17(12) | Yes |
| 62/M^1^ | B2 |  | 20/8 | 51/408 | *Streptococcus pneumoniae* | Yes | Yes | 39(30) | Yes |
| 39/F^2^ | A2A |  | 12/3 | 0.1/38 | No | No | No | 7(4) | Yes |
| 76/F^2^ | B2 |  | 9/2 | 10,5/300 | No | No | No | 48(27) | Yes |
| 75/F^2^ | B2 | OSA^4^ | 17/11 | 0.6/13 | No | No | Yes | 14(9) | Yes |
| 83/F^2^ | A2A | CHF^5^ | 13/6 | 18.2/163 | *Streptococcus pneumoniae* | No | Yes | 14(5) | Yes |
| 42/M^1^ | B1 |  | 5/8 | 8.9/231 | *Streptococcus pneumoniae* | No | Yes | 16(6) | Yes |
| 67/M^1^ | B1 |  | 13/3 | 0.16/70 | No | No | No | 18(11) | Yes |
| 77/M^1^ | B2 |  | 34/14 | 0.16/97 | No | No | Yes | 3(3) | No |
| 54/M^1^ | _ |  | 13/11 | 0.12/35 | Parainfluenza 3 | Yes | Yes | 11(7) | Yes |
| 49/F^2^ | B1 | COPD^3^ | 11/6 | 0.03/15 | No | No | No | 13(5) | Yes |
| 81/F^2^ | B1 |  | 19/7 | 0.09/47 | No | No | No | 15(5) | Yes |
| 14/F^2^ | A2B | Asthma | 8/2 | 0.12/170 | No | No | No | 10(6) | Yes |
| 38/M^1^ | B2 |  | 8/7 | 0.8/187 | No | Yes | Yes | 31(25) | Yes |
| 62/M^1^ | B1 | Dilated Cardiomyopathy | 12/3 | 0.54/62.7 | No | No | No | 14(10) | Yes |
| 76/M^1^ | B1 | COPD^3^ | 24/8 | 71.8/324 | *Streptococcus pneumoniae* | No | Yes | 9(5) | Yes |
| 47/M^1^ | B1 | Asthma | 12/8 | 0.29/133 | No | Yes | Yes | 36(29) | Yes |
| 62/M^1^ | B2 | Enolism | 16/1 | 20.7/51602 | No | No | No | 9(5) | Yes |
| 84/F^2^ | B1 |  | 26/11 | 8.42/37.5 | *Streptococcus pneumoniae* | No | Yes | 25(10) | Yes |

^1^M: Male; ^2^F: Female; ^3^COPD: Chronic obstructive pulmonary disease; ^4^OSA: Obstructive sleep apnea; ^5^CHF: Congestive heart failure.
