# Supplementary material for: Human metapneumovirus as cause of severe community-acquired pneumonia in adults: insights from a ten-year molecular and epidemiological analysis
Source: Ann Intensive Care. 2019 Jul 24;9:86. doi: 10.1186/s13613-019-0559-y (PMC6656825; doi:10.1186/s13613-019-0559-y)
Supplement: Supplementary file 2 — Additional file 2: Table S2. Main characteristics of immunosuppressed adult patients admitted to the Intensive Care Unit due to a severe community-acquired pneumonia associated with human metapneumovirus infection (Guipuzcoa, Basque Country, Spain, 2007–2017). [file 13613_2019_559_MOESM2_ESM.docx]

Additional file 2: Table S2: Main characteristics of immunosuppressed adult patients admitted to the Intensive Care Unit due to a Severe community-acquired pneumonia associated to human metapneumovirus infection (Guipuzcoa, Basque Country, Spain, 2007–2017)

| Age/Sex | Genotype | Comorbidities | APACHE II /SOFA | PCT(ng/ml) CRP(mg/dl) | Coinfection | ARDS | SHOCK | Hospital (ICU) stay | Survival at discharge |
| --- | --- | --- | --- | --- | --- | --- | --- | --- | --- |
| 30/M^1^ | A2B | Non-Hodgkin linphoma | 20/13 | 2.07/458 | CMV^4^ | Yes | Yes | 26(26) | No |
| 75/M^1^ | A2B | Multiple myeloma | 23/7 | 7.9/118 | No | Yes | Yes | 39(16) | Yes |
| 62/M^1^ | A2A | Heart transplant | 17/5 | 4/84.1 | *Streptococcus pneumoniae* | No | No | 9(5) | Yes |
| 77/F^2^ | B1 | Chemotherapy | 22/11 | 1.4/227 | No | Yes | Yes | 5(5) | No |
| 60/F^2^ | B1 | SLE^3^ | 31/10 | 1.2/102 | No | No | Yes | 11(9) | No |
| 52/M^1^ | B1 | Pancreas and kidney transplant | 13/3 | 77/500 | No | No | No | 10(6) | Yes |
| 61/M^1^ | A2B | Kidney transplant | 21/5 | 57.5/480 | *Streptococcus pneumoniae* | No | No | 9(3) | Yes |

^1^M: Male; ^2^F: Female; ^3^SLE: Systemic lupus erythematosus. ^4^CMV: Cytomegalovirus.
